# Status of the new genera in Gistel’s “Die Insecten-Doubletten aus der Sammlung des Herrn Grafen Rudolph von Jenison Walworth” issued in 1834

**DOI:** 10.3897/zookeys.698.14913

**Published:** 2017-09-18

**Authors:** Yves Bousquet, Patrice Bouchard

**Affiliations:** 1 Canadian National Collection of Insects, Arachnids and Nematodes, Agriculture and Agri-Food Canada, 960 Carling Avenue, Ottawa, Ontario, K1A 0C6, Canada

**Keywords:** Beetles, nomenclature, Gistel, genus-group names, type species, *nomen oblitum*, *nomen protectum*

## Abstract

All new genus-group names included in Gistel’s list of Coleoptera from the collection of Count Rudolph von Jenison Walwort, published in 1834, are recorded. For each of these names, the originally included available species are listed and for those with at least one available species included, the type species and current status are provided.

The following new synonymies are proposed [valid names in brackets]: *Auxora* [*Nebria* Latreille, 1806; Carabidae], *Necrotroctes* [*Velleius* Leach, 1819; Staphylinidae], *Epimachus* [*Ochthephilum* Stephens, 1829; Staphylinidae], *Ocys* [*Anaulacaspis* Ganglbauer, 1895; Staphylinidae], *Hydatobia* [*Autalia* Samouelle, 1819; Staphylinidae], *Hedonius* [*Pyrophorus* Billberg, 1820; Elateridae], *Charmionus* [*Chalcolepidius* Eschscholtz, 1829; Elateridae], *Lamprias* [*Alaus* Eschscholtz, 1829; Elateridae], *Trypheus* [*Aeolus* Eschscholtz, 1829; Elateridae], *Antiphus* [*Cardiorhinus* Eschscholtz, 1829; Elateridae], *Phyletus* [*Lygistopterus* Dejean, 1833; Lycidae], *Phyllogaster* [*Lucidota* Laporte, 1833; Lampyridae], *Pyrrhigius* [*Phosphaenus* Laporte, 1833; Lampyridae], *Erota* [*Luciola* Laporte, 1833; Lampyridae], *Oxypterus* [*Aspisoma* Laporte, 1833; Lampyridae], *Phyllophagus* [*Chauliognathus* Hentz, 1830; Cantharidae], *Epaphius* [*Astylus* Laporte, 1836; Melyridae], *Isomerus* [*Choleva* Latreille, 1797; Leiodidae], *Berecyntha* [*Aulacochilus* Chevrolat, 1836; Erotylidae], *Geophilus* [*Psammodius* Fallén, 1807; Scarabaeidae], *Ceraunus* [*Golofa* Hope, 1837; Scarabaeidae], *Atrimedeus* [*Pentodon* Hope, 1837; Scarabaeidae], *Eupalus* [*Temnorhynchus* Hope, 1837; Scarabaeidae], *Polycarmes* [*Anoxia* Laporte, 1832; Scarabaeidae], *Acidota* [*Amphicoma* Latreille, 1807; Glaphyridae], *Cecrops* [*Mylaris* Pallas, 1781; Tenebrionidae], *Pythonissus* [*Zophobas* Dejean, 1834; Tenebrionidae], *Physignathus* [*Cymatothes* Dejean, 1834; Tenebrionidae], *Pelops* [*Prionychus* Solier, 1835; Tenebrionidae], *Accantosomus* [*Semiotus* Eschscholtz, 1829; Elateridae].

The type species of the following genus-group taxa are proposed: *Ocys* [*Aleochara nigra* Gravenhorst, 1802; Staphylinidae], *Hydatobia* [*Staphylinus impressus* Olivier, 1795; Staphylinidae], *Hedonius* [*Elater noctilucus* Linnaeus, 1758; Elateridae], *Charmionus* [*Elater porcatus* Linnaeus, 1767; Elateridae], *Epaphius* [*Dasytes variegatus* Germar, 1823; Melyridae], *Geophilus* [*Scarabeus asper* Fabricius, 1775; Scarabaeidae], *Atrimedeus* [*Scarabaeus punctatus* Villers, 1789; Scarabaeidae], *Polycarmes* [*Melolontha villosa* Fabricius, 1781; Scarabaeidae], *Cecrops* [*Tenebrio gigas* Linnaeus, 1763; Tenebrionidae], *Pythonissus* [*Helops morio* Fabricius, 1777; Tenebrionidae], *Ceratades* [*Cerambyx sutor* Linnaeus, 1758; Cerambycidae].

The following genus-group names are declared *nomina oblita* [*nomina protecta* in square brackets]: *Berecyntha* [*Aulacochilus* Chevrolat, 1836; Erotylidae], *Ceraunus* [*Golofa* Hope, 1837; Scarabaeidae], *Atrimedeus* [*Pentodon* Hope, 1837; Scarabaeidae], *Eupalus* [*Temnorhynchus* Hope, 1837; Scarabaeidae], *Pelops* [*Prionychus* Solier, 1835; Tenebrionidae].

## Introduction

Gistel [1809–1873], also spelled Gistl, was an enigmatic and controversial man. Evenhuis (2016) provided valuable biographical information on this personage who sometimes used pseudonyms (i.e., G. Tilesius and Garduus). He was described as vainglorious, sensationalistic, and sometimes superficial and inaccurate (Strand 1919: 125) and accused of being a cheater and a liar. He often changed specific names, sometimes just to be able to dedicate them to his friends (Mannerheim 1838: 207). He also proposed many new generic names, often in groups out of his area of expertise, most without descriptions. Although they were largely ignored by Gistel’s contemporaries, some of these genus-group names turned out to be nomenclaturally available when he listed at least one available species names within them (ICZN 1999: Article 12.2.5).

One of Gistel’s publications with several new generic names proposed without description was issued in 1834 under the title “*Die Insecten-Doubletten aus der Sammlung des Herrn Grafen Rudolph von Jenison Walworth*.” The 36-page booklet is a list of duplicate beetles in the collection of Count Rudolph von Jenison Walworth [1778–1835] of Regensburg offered for purchase or exchange (Gistel 1834b). Several of the names included in this work have been assessed nomenclaturally in recent years but others have not. To add to the confusion, the status of some genus-groups names has been incorrectly interpreted. The objective of this paper is to summarize the nomenclatural status of all new genus-group names proposed in this work.

### Presentation

The presentation of the information relevant to the new genus-group names is similar to that used for the names in Dejean’s catalogue (Bousquet and Bouchard 2013). Under each new genus, the following information is provided: a list of originally included available species, the type species of the genus, and its current status. Genus- and species-group names are given in the order they appear in Gistel’s work.

The earliest known date of publication for the publication of Gistel’s booklet is 23 September 1834 (Kielmeyer and Jäger 1835: 101). All available species-group taxa published before that date and included in Gistel’s booklet are considered to be originally included in the new genus-group names. In addition, all genus-group names issued before that date take precedence over Gistel’s names.

### Note on some generic names in Gistel (1834b)

One genus mentioned in Gistel’s work is *Necrobius* (Gistel 1834b: 14), a junior objective synonym of *Necrophilus* Latreille, 1829 [Agyrtidae: Necrophilinae]. This genus was however first made available by Gistel (1834a: 147) in the third *Heft* (pp. 129–190) of the first volume of his journal “*Faunus. Zeitschrift für Zoologie und vergleichende Anatomie*.” This *Heft* was issued before 16 August 1834 as it was recorded in *Die Bayer’sche Landbötin* No 98 (p. 802) on this date. *Necrobius* is therefore excluded from the list below.

In Gistel’s booklet, the generic names *Cychramus* (p. 15) and *Heliophilus* (p. 21) are credited to himself. It is quite obvious that Gistel made lapses regarding authorship of these names since they were established for the same species by Kugelann (1794) and Dejean (1821) respectively and used subsequently by several authors prior to Gistel (1834b). These names are therefore also excluded from the list below.

The genus *Macropterum* is credited to Gistel (1834b: 9) in the literature (e.g., Blackwelder 1952: 229; Herman 2001: 636, Gamarra and Outerelo 2009: 209; Schülke and Smetana 2015a: 357). However, the name was first made available in Gistel (1829: 37). The genus is considered valid but listed as a *nomen dubium* [Staphylinidae: Proteininae: Proteinini] by Schülke and Smetana (2015a: 357).

The genus-group name *Hammionus* was first used as valid by Dahl (1823: 22) but this work was suppressed by the International Commission on Zoological Nomenclature in Opinion 710 (ICZN 1964). It was subsequently listed by Latreille (1829: 456) as a synonym of *Campylus* Fischer von Waldheim, 1821 and by Gistel (1834b: 11) as a valid name. Following Article 11.6 (ICZN 1999), the name must be attributed to Latreille (1829). The species first directly associated with the name are those mentioned in Gistel (1834b: 11). The type species is *Elater bructeri* Panzer, 1795 (= *Elater aeneoniger* DeGeer, 1774) by subsequent designation (Ruiz 1996: 109). *Hammionus* Latreille, 1829 is a junior homonym of *Hammionus* Charpentier, 1825 (Coleoptera: Elateridae) and a senior objective synonym of *Pheletes* Kiesenwetter, 1858 [Elateridae: Dendrometrinae: Dendrometrini: Dendrometrina] (*fide* Cate 2007: 166).

Alonso-Zarazaga and Lyall (1999: 27) treated *Camplirhynchus* Gistel (1834b: 27, as “Camplirhynchus Meg.”) as an available name. However, we believe it is an incorrect subsequent spelling of *Campylirhynchus* Dejean (1821: 84, as “Campylirhynchus *Meg.*”) since both names were used, in part, for the same species.

## List of new genus-group names

***Auxora* Gistel, 1834b: 3**

Originally included available species: *Nebria heydenii* Dejean, 1831 (as “heidenii *Par.* Ins. Jon.”).

Type species: *Nebria heydenii* Dejean, 1831 by monotypy.

Current status: junior synonym of *Nebria* Latreille, 1806 [Carabidae: Nebriinae: Nebriini] (**new synonymy**).

***Lymnaeus*Gistel 1834b: 7**

Originally included available species: *Dytiscus fuscus* Linnaeus, 1758 (as “fuscus *Fab.*”); *Dytiscus striatus* Linnaeus, 1758 (as “striatus *id.* [Fab.]”); *Dytiscus dolabratus* Paykull, 1798 (as “dolobratus *Pay.*”).

Type species: *Dytiscus dolabratus* Paykull, 1798 by subsequent designation (Nardi and Nilsson 2006: 35).

Current status: junior subjective synonym of *Colymbetes* Clairville, 1806 [Dytiscidae: Colymbetinae: Colymbetini] (*fide* Nardi and Nilsson 2006: 35).

Note: Gistel’s name is not a junior homonym of Cuvier’s “*Lymnaeus*” since this name is an incorrect subsequent spelling of *Lymnaea* Lamarck, 1799 (Mollusca, Gastropoda) not in prevailing usage.

***Hydatobius*Gistel 1834b: 7**

Originally included available species: *Dytiscus oblongus* Illiger, 1801.

Type species: *Dytiscus oblongus* Illiger, 1801 (= *Dytiscus haemorrhoidalis* Fabricius, 1787) by monotypy.

Current status: junior objective synonym of *Liopterus* Dejean, 1833 [Dytiscidae: Copelatinae] (*fide* Nardi and Nilsson 2006: 35).

***Pelidus* Gistel, 1834b: 7**

Originally included available species: *Dytiscus notatus* Bergsträsser, 1778 (as “notatus *Fab.*”); *Dytiscus agilis* Fabricius, 1792; *Dytiscus adspersus* Panzer, 1796 (as “adspersus *id.* [Fab.]”).

Type species: *Dytiscus agilis* Fabricius, 1792 (=*Dytiscus haemorrhoidalis* Fabricius, 1787) by subsequent designation (Nardi and Nilsson 2006: 35).

Current status: junior subjective synonym of *Liopterus* Dejean, 1833 [Dytiscidae: Copelatinae] (*fide* Nardi and Nilsson 2006: 35).

***Hydronectes* Gistel, 1834b: 8**

Originally included available species: *Gyrinus villosus* O.F. Müller, 1776 (as “villosus *Fab.*”).

Type species: *Gyrinus villosus* O.F. Müller, 1776 by monotypy.

Current status: junior objective synonym of *Orectochilus* Dejean, 1833 [Gyrinidae: Gyrininae: ] (*fide* Nardi and Nilsson 2006: 35).

***Necrotroctes* Gistel, 1834b: 8**

Originally included available species: *Staphylinus dilatatus* Fabricius, 1787.

Type species: *Staphylinus dilatatus* Fabricius, 1787 by monotypy.

Current status: junior objective synonym of *Velleius* Leach, 1819 [Staphylinidae: Staphylininae: Staphylinini: Quediina, *Quedius* Stephens] (**new synonymy**).

***Aemulus* Gistel, 1834b: 8**

Originally included available species: *Staphylinus lateralis* Gravenhorst, 1802; *Staphylinus fuliginosus* Gravenhorst, 1802; *Staphylinus molochinus* Gravenhorst, 1806; *Staphylinus nitidus* Fabricius, 1787; *Staphylinus scitus* Gravenhorst, 1806; *Staphylinus impressus* Panzer, 1796 (as “impressus *Gra.*”); *Staphylinus boops* Gravenhorst, 1802.

Type species: *Staphylinus fuliginosus* Gravenhorst, 1802 by subsequent designation (Blackwelder 1952: 40).

Current status: junior subjective synonym of *Quedius* Stephens, 1829 [Staphylinidae: Staphylininae: Staphylinini: Quediina] (*fide* Schülke and Smetana 2015b: 1067).

***Laxobates* Gistel, 1834b: 8**

Originally included available species: *Staphylinus coenosus* Gravenhorst, 1806; *Staphylinus cyanipennis* Fabricius, 1792 (as “cyanipennis *Gra.*”); *Staphylinus splendens* Fabricius, 1792; *Staphylinus laminatus* Creutzer, 1799 (as “laminatus *Gra.*”); *Staphylinus aeneus* Rossi, 1790 (as “aeneus *id.* [*Gra.*]”); *Staphylinus metallicus* Gravenhorst, 1802; *Staphylinus cephalotes* Gravenhorst, 1802; *Staphylinus decorus* Gravenhorst, 1802; *Staphylinus politus* Linnaeus, 1758 (as “politus *Fab.*”); *Staphylinus atratus* Gravenhorst, 1802; *Staphylinus carbonarius* Gravenhorst, 1802; *Staphylinus ebeninus* Gravenhorst, 1802 (as “ebeneninus *Gra.*”); *Staphylinus varians* Paykull, 1789 (as “varians *Gyll.*”); *Staphylinus varius* Gyllenhal, 1810; *Staphylinus immundus* Gravenhorst, 1806 (as “immundus *Gyll.*”); *Staphylinus fuscus* Gravenhorst, 1802; *Staphylinus sanguinolentus* Gravenhorst, 1802; *Staphylinus bipustulatus* Linnaeus sensu Panzer, 1795 (as “bipustulatus *Fab.*”) (= *Staphylinus cruentatus* Gmelin, 1789); *Staphylinus bimaculatus* Gravenhorst, 1802; *Staphylinus fulvipes* Fabricius, 1792 (as “fulvipes *Gra.*”); *Staphylinus tenuis* Fabricius, 1792 (as “tenuis *id.* [Gra.]”); *Staphylinus fimetarius* Gravenhorst, 1802; *Staphylinus agilis* Gravenhorst, 1806; *Staphylinus splendidulus* Gravenhorst, 1802; *Staphylinus aterrimus* Gravenhorst, 1802.

Type species: *Staphylinus splendens* Fabricius, 1792 by subsequent designation (Blackwelder 1952: 212).

Current status: junior objective synonym of *Philonthus* Stephens, 1832 [Staphylinidae: Staphylininae: Staphylinini: Philonthina] (*fide* Schülke and Smetana 2015b: 1034).

***Epimachus* Gistel, 1834b: 8**

Originally included available species: *Paederus fracticornis* Paykull, 1800 (as “fracticornis *Gra.*”).

Type species: *Paederus fracticornis* Paykull, 1800 by monotypy.

Current status: junior homonym of *Epimachus* Cuvier, 1817 (Aves); junior objective synonym of *Ochthephilum* Stephens, 1829 [Staphylinidae: Paederinae: Cryptobiina] (**new synonym**).

Note: Gistel included one species “fracticornis *Gra.* Bav.” Blackwelder (1952: 150) referred the species to *Staphylinus fracticornis* Paykull, 1790 [a senior synonym of *Bledius gallicus* (Gravenhorst, 1806), see Schülke and Smetana (2015b: 763)] but this species was placed in the genus *Dicarenus* by Gistel (1834b: 9).

***Sepedomorphus* Gistel, 1834b: 9**

Originally included available species: *Staphylinus fragilis* Gravenhorst, 1802; *Staphylinus orbiculatus* Paykull, 1789 (as “orbiculatus *Fab.*”).

Type species: *Staphylinus orbiculatus* Paykull, 1789 by subsequent designation (Blackwelder 1952: 350).

Current status: junior objective synonym of *Rugilus* Leach, 1819 [Staphylinidae: Paederinae: Stilicina] (*fide* Schülke and Smetana 2015b: 998).

***Dicarenus* Gistel, 1834b: 9**

Originally included available species: *Staphylinus tricornis* Herbst, 1784 (as “tricornis *Gra.*”); *Oxytelus pallipes* Gravenhorst, 1806 (“pallipes *Gyll.*”); *Staphylinus fracticornis* Paykull, 1790 (as “fracticornis *Gyl.*”); *Oxytelus talpa* Gyllenhal, 1810; *Staphylinus arenarius* Paykull, 1800 (as “arenarius *id.* [Gyll.]”).

Type species: *Staphylinus arenarius* Paykull, 1800 (= *Bledius fergussoni* Joy, 1912) by subsequent designation (Blackwelder 1952: 124).

Current status: valid subgenus of *Bledius* Leach, 1819 [Staphylinidae: Oxytelinae: Blediini] (*fide* Schülke and Smetana 2015a: 760).

***Batychrus* Gistel, 1834b: 9**

Originally included available species: *Oxytelus corticinus* Gravenhorst, 1806.

Type species: *Oxytelus corticinus* Gravenhorst, 1806 by monotypy.

Current status: junior objective synonym of *Trogophloeus* Mannerheim, 1830 [Staphylinidae: Oxytelinae: Oxytelini, *Carpelimus* Leach] (*fide* Schülke and Smetana 2015a: 785).

***Helobium* Gistel, 1834b: 9**

Originally included available species: *Staphylinus crenatus* Fabricius, 1792.

Type species: *Staphylinus crenatus* Fabricius, 1792 by monotypy.

Current status: junior homonym of *Helobium* Leach, 1815 (Coleoptera: Carabidae); junior objective synonym of *Acidota* Stephens, 1829 [Staphylinidae: Omaliinae: Anthophagini] (*fide* Schülke and Smetana 2015a: 304).

***Psyllius* Gistel, 1834b: 9**

Originally included available species: *Staphylinus depressus* Paykull, 1789 (as “depressus *Gyll.*”).

Type species: *Staphylinus depressus* Paykull, 1789 by monotypy.

Current status: junior objective synonym of *Megarthrus* Stephens, 1829 [Staphylinidae: Proteininae: Proteinini] (*fide* Schülke and Smetana 2015a: 353).

***Bolitoglyphus* Gistel, 1834b: 9**

Originally included available species: *Staphylinus atricapillus* Fabricius, 1775; *Staphylinus striatus* Olivier, 1795 (as “striatus *Gra.*”); *Staphylinus analis* Fabricius sensu Gravenhort, 1802 (as “analis *Gra.*”) (= *Megacronus castaneus* Stephens, 1832); *Staphylinus merdarius* Fabricius, 1775; *Oxyporus melanocephalus* Fabricius, 1792 (as “melanocephalus *Gra.*”)

Type species: *Megacronus castaneus* Stephens, 1832 fixed by Schülke (1999: 982) (under Article 70.3 of the Code), misidentified as *Staphylinus analis* Fabricius, 1787 in the original designation by Blackwelder (1952: 82).

Current status: junior objective synonym of *Bolitobius* Leach, 1819 [Staphylinidae: Tachyporinae: Mycetoporini] (*fide* Schülke and Smetana 2015a: 456).

***Leichotes* Gistel, 1834b: 9**

Originally included available species: *Tachinus punctus* Gravenhorst, 1806; *Tachinus lepidus* Gravenhorst, 1806; *Tachinus splendidus* Gravenhorst, 1806.

Type species: *Tachinus splendidus* Gravenhorst, 1806 by subsequent designation (Blackwelder 1952: 212).

Current status: junior objective synonym of *Ischnosoma* Stephens, 1829 [Staphylinidae: Tachyporinae: Mycetoporini] (*fide* Schülke and Smetana 2015a: 459). Genus placed on the Official Index of Rejected and Invalid Generic Names in Zoology (ICZN 1993).

***Hesperophilus* Gistel, 1834b: 9**

Originally included available species: *Lomechusa dentata* Gravenhorst, 1806.

Type species: *Lomechusa dentata* Gravenhorst, 1806 by monotypy.

Current status: junior homonym of *Hesperophilus* Stephens, 1829 (Coleoptera: Staphylinidae); junior objective synonym of *Dinarda* Leach, 1819 [Staphylinidae: Aleocharinae: Oxypodini: Dinardina] (*fide* Schülke and Smetana 2015a: 681).

***Agaricola* Gistel, 1834b: 10**

Originally included available species: *Staphylinus canaliculatus* Fabricius, 1787.

Type species: *Staphylinus canaliculatus* Fabricius, 1787 by monotypy.

Current status: junior objective synonym of *Drusilla* Leach, 1819 [Staphylinidae: Aleocharinae: Lomechusini: Myrmedoniina] (*fide* Schülke and Smetana 2015a: 665).

***Ocys* Gistel, 1834b: 10**

Originally included available species: *Aleochara obscura* Gravenhorst, 1802; *Aleochara nigra* Gravenhorst, 1802; *Aleochara picea* Gravenhorst, 1802.

Type species: *Aleochara nigra* Gravenhorst, 1802 by **present designation**.

Current status: junior homonym of *Ocys* Stephens, 1828 (Coleoptera: Carabidae); senior objective synonym of *Anaulacaspis* Ganglbauer, 1895 [Staphylinidae: Aleocharinae: Falagriini] (**new synonym**).

***Hydatobia* Gistel, 1834b: 10**

Originally included available species: *Aleochara rivularis* Gravenhorst, 1802; *Staphylinus impressus* Olivier, 1795 (“as impressa *id.* [Gra.]”).

Type species: *Staphylinus impressus* Olivier, 1795 by **present designation**.

Current status: junior objective synonym of *Autalia* Samouelle, 1819 [Staphylinidae: Aleocharinae: Autaliini] (**new synonym**).

***Charytonia* Gistel, 1834b: 10**

Originally included available species: *Buprestis chrysis* Fabricius, 1775; *Buprestis sternicornis* Linnaeus, 1758 (as “sternicornis *id.* [Fab.]”).

Type species: *Buprestis chrysis* Fabricius, 1775 by subsequent designation (Bilý 2001: 148).

Current status: junior objective synonym of *Sternocera* Eschscholtz, 1829 [Buprestidae: Julodinae] (*fide* Bellamy 2008a: 85).

***Phyllis* Gistel, 1834b: 10**

Originally included available species: *Buprestis fascicularis* Linnaeus, 1758 (as “fascicularis *Fab.*”); *Buprestis hirta* Linnaeus, 1758 (as “hirta *id.* [Fab.]”); *Buprestis vulnerata* Perty, 1830.

Type species: *Buprestis fascicularis* Linnaeus, 1758 by subsequent designation (Bellamy 2003: 11).

Current status: junior homonym of *Phyllis* Robineau-Desvoidy, 1830 (Diptera, now a *nomen oblitum* following Evenhuis et al. 2010: 137); junior objective synonym of *Julodis* Eschscholtz, 1829 [Buprestidae: Julodinae] (*fide* Bellamy 2008a: 46).

***Andromeda* Gistel, 1834b: 10**

Originally included available species: *Buprestis ornata* Fabricius, 1775; *Buprestis pulcra* Fabricius, 1792; *Buprestis octodecimguttata* Piller & Mitterpacher, 1783 (as “octodecimguttata *Her.*”); *Buprestis taeniata* Fabricius, 1787; *Buprestis cylindrica* Fabricius, 1775; *Buprestis gibbosa* Olivier, 1790 (as “gibbosa *id.* [Fab.]”).

Type species: *Buprestis ornata* Fabricius, 1775 by subsequent designation (Bellamy 2003: 10).

Current status: junior subjective synonym of *Acmaeodera* Eschscholtz, 1829 [Buprestidae: Polycestinae: Acmaeoderini] (*fide* Bellamy 2008a: 148).

***Pyranthe* Gistel, 1834b: 10**

Originally included available species: *Buprestis ocellata* Fabricius, 1775; *Buprestis vittata* Fabricius, 1775; *Buprestis fulgida* Olivier, 1790 (as “fulgida *id.* [Fab.]”).

Type species: *Buprestis vittata* Fabricius, 1775 by subsequent designation (Bilý 2001: 149).

Current status: valid subgenus of *Chrysochroa* Dejean, 1833 [Buprestidae: Chrysochroinae: Chrysochroini] (*fide* Bellamy 2008a: 429).

***Phalantha* Gistel, 1834b: 10**

Originally included available species: *Buprestis scabra* Fabricius, 1775.

Type species: *Buprestis scabra* Fabricius, 1775 by monotypy.

Current status: junior objective synonym of *Steraspis* Dejean, 1833 [Buprestidae: Chrysochroinae: Chrysochroini] (*fide* Bellamy 2008a: 461).

***Archonta* Gistel, 1834b: 10**

Originally included available species: *Buprestis gigantea* Linnaeus, 1758 (as “gigantea *Fab.*”).

Type species: *Buprestis gigantea* Linnaeus, 1758 by monotypy.

Current status: junior homonym of *Archonta* de Montfort, 1810 (Mollusca); junior objective synonym of *Euchroma* Dejean, 1833 [Buprestidae: Chrysochroinae: : ] (*fide* Bellamy 2008a: 572).

***Polydora* Gistel, 1834b: 10**

Originally included available species: *Buprestis equestris* Fabricius, 1792; *Buprestis amoena* Kirby, 1819; *Buprestis speculigera* Perty, 1830 (as “speculifera *Pty.*”).

Type species: *Buprestis amoena* Kirby, 1819 by subsequent designation (Bellamy 2003: 11).

Current status: junior homonym of *Polydora* Bosc, 1802 (Polychaeta) and *Polydora* Oken, 1816 (Annelida); junior objective synonym of *Conognatha* Eschscholtz, 1829 [Buprestidae: Buprestinae: ] (*fide* Bellamy 2008b: 1217).

***Hiperantha* Gistel, 1834b: 10**

Originally included available species: *Buprestis interrogationis* Klug, 1825.

Type species: *Buprestis interrogationis* Klug, 1825 by monotypy.

Current status: valid genus [Buprestidae: Buprestinae: ] (*fide* Bellamy 2008b: 1253).

***Caloptera* Gistel, 1834b: 10**

Originally included available species: *Buprestis cariosa* Pallas, 1776 (as “cariosa *Fab.*”); *Buprestis tenebrionis* Linnaeus, 1761 (as “Tenebrionis *id.* [Fab.]”); *Buprestis tenebricosa* Olivier, 1790 (as “tenebricosa *id.* [Fab.]”).

Type species: *Buprestis tenebrionis* Linnaeus, 1761 by subsequent designation (Bellamy 2003: 10).

Current status: junior homonym of *Caloptera* Guérin-Méneville, 1831 (Diptera); junior subjective synonym of *Capnodis* Eschscholtz, 1829 [Buprestidae: Chrysochroinae: Dicercini: Dicercina] (*fide* Bellamy 2008b: 951).

***Argante* Gistel, 1834b: 10**

Originally included available species: *Buprestis moesta* Fabricius, 1792; *Bupestris aenea* Linnaeus, 1761; *Buprestis berolinensis* Herbst, 1779 (as “berolinensis *id.* [Fab.]”); *Buprestis calcarata* Fabricius, 1801; *Buprestis alni* Fischer von Waldheim, 1824 (as “Var. *Alni Meg.*”); *Buprestis acuminata* Pallas, 1781 (as “acuminata *Fab.*”).

Type species: *Buprestis moesta* Fabricius, 1792 by subsequent designation (Richter 1952: 108).

Current status: junior subjective synonym of *Dicerca* Eschscholtz, 1829 [Buprestidae: Chrysochroinae: Dicercini: Dicercina] (*fide* Bellamy 2008b: 974).

Note: *Neoargante*, proposed by Nelson (2006: 373), is an unnecessary replacement name for *Argante* Gistel, 1834.

***Gymnota* Gistel, 1834b: 10**

Originally included available species: *Buprestis rustica* Linnaeus, 1758 (as “rustica *Fab.*”); *Buprestis punctata* Fabricius, 1787; *Buprestis flavopuncta* DeGeer, 1774 (as “flavomaculata *id.* [Fab.]”); *Buprestis octoguttata* Linnaeus, 1758 (as “octoguttata *id.* [Fab.]”).

Type species: *Buprestis rustica* Linnaeus, 1758 by subsequent designation (Holyński 1993: 28).

Current status: *Gymnota* Gistel, 1834 is a senior homonym of *Gymnota* Gistel, 1847 (Coleoptera: Chrysomelidae), an unjustified emendation for *Lina* Latreille, 1829; junior objective synonym of *Ancylocheira* Eschscholtz, 1829 [Buprestidae: Buprestinae: Buprestini: Buprestina] (*fide* Bellamy 2008b: 1025).

Note: Fabricius (1801: 193) incorrectly wrote DeGeer’s name “*flauomaculata*” instead of *flavopuncta*.

***Euptera* Gistel, 1834b: 10**

Originally included available species: *Buprestis angularis* Dalman, 1817 (as “angularis *Schö.*”).

Type species: *Buprestis angularis* Dalman, 1817 by monotypy.

Current status: junior objective synonym of *Pelecopselaphus* Solier, 1833 (= *Pristiptera* Dejean, 1833; see Bousquet and Bouchard 2013: 22) [Buprestidae: Chrysochroinae: : ] (*fide* Bellamy 2008a: 586).

Note: *Euptera* Gistel, 1834 is a senior homonym of *Euptera* Staudinger, 1891, currently a valid genus in Lepidoptera (Nymphalidae). A new name is needed for the lepidopteran generic name.

***Lycaste* Gistel, 1834b: 10**

Originally included available species: *Buprestis porcata* Fabricius, 1775.

Type species: *Buprestis porcata* Fabricius, 1775 by monotypy.

Current status: junior objective synonym of *Polycesta* Dejean, 1833 [Buprestidae: Polycestinae: Polycestini: ] (*fide* Bellamy 2008a: 365).

***Amblis* Gistel, 1834b: 10**

Originally included available species: *Buprestis chrysostigma* Linnaeus, 1758 (as “chrysostigma *Fab.*”); *Buprestis affinis* Fabricius, 1794.

Type species: *Buprestis chrysostigma* Linnaeus, 1758 by subsequent designation (Bellamy 2003: 10).

Current status: junior objective synonym of *Chrysobothris* Eschscholtz, 1829 [Buprestidae: Buprestinae: Chrysobothrini] (*fide* Bellamy 2008c: 1587).

***Chrysodora* Gistel, 1834b: 11**

Originally included available species: *Buprestis antiqua* Illiger, 1803; *Buprestis geminata* Illiger, 1803.

Type species: *Buprestis antiqua* Illiger, 1803 by subsequent designation (Bellamy 2003: 10).

Current status: junior objective synonym of *Sphenoptera* Dejean, 1833 [Buprestidae: Chrysochroinae: Sphenopterini] (*fide* Bellamy 2008b: 635).

***Xanthus* Gistel, 1834b: 11**

Originally included available species: *Elater obscurus* Linnaeus, 1758 (as “obscurus *Fab.*”); *Elater fulvipes* Herbst, 1806; *Elater castanipes* Paykull, 1800; *Elater niger* Linnaeus sensu Fabricius, 1792 (as “niger *Fab.*”).

Type species: *Elater niger* Linnaeus sensu Fabricius, 1792 (= *Elater punctolineatus* Pelerin, 1829) by subsequent designation (Ruiz 1996: 167).

Current status: junior subjective synonym of *Melanotus* Eschscholtz, 1829 [Elateridae: Elaterinae: Melanotini] (*fide* Cate 2007: 141).

Note: Ruiz (1996: 167) selected “*Elater niger* Fabricius, 1792” as type species but Fabricius (1792: 221) referred the species to “Linn. Syst. nat. 2. 656. 36. Fn. Sv. 743.”

***Hedonius* Gistel, 1834b: 11**

Originally included available species: *Elater noctilucus* Linnaeus, 1758 (as noctilucus *Fab.*”); *Elater phosphoreus* Linnaeus, 1758; *Elater ignitus* Fabricius, 1787; *Elater luminosus* Illiger, 1807.

Type species: *Elater noctilucus* Linnaeus, 1758 by **present designation**.

Current status: junior objective synonym of *Pyrophorus* Billberg, 1820 [Elateridae: Agrypninae: Pyrophorini] (**new synonym**).

***Charmionus* Gistel, 1834b: 11**

Originally included available species: *Elater sulcatus* Fabricius, 1777; *Elater porcatus* Linnaeus, 1767 (as “porcatus *id.* [Fab.]”); *Elater striatus* Linnaeus, 1767 (as “striatus *id.* [Fab.]”); *Elater virens* Fabricius, 1787.

Type species: *Elater porcatus* Linnaeus, 1767 by **present designation**.

Current status: junior subjective synonym of *Chalcolepidius* Eschscholtz, 1829 [Elateridae: Agrypninae: Hemirhipini] (**new synonym**).

***Lamprias* Gistel, 1834b: 11**

Originally included available species: *Elater oculatus* Linnaeus, 1758 (as “oculatus *Fab.*”).

Type species: *Elater oculatus* Linnaeus, 1758 by monotypy.

Current status: junior homonym of *Lamprias* Dejean, 1825 (Coleoptera: Carabidae); junior objective synonym of *Alaus* Eschscholtz, 1829 [Elateridae: Agrypninae: Hemirhipini] (**new synonym**).

***Gripus* Gistel, 1834b: 11**

Originally included available species: *Elater rufus* Fabricius, 1792; *Elater rhombeus* Olivier, 1790; *Elater undulatus* DeGeer, 1774 (as “undulatus *Pay.*”); *Elater scrutator* Herbst, 1806; *Elater testaceus* Fabricius sensu Paykull, 1800 (= *Elater scrutator* Herbst, 1806); *Elater hirtus* Herbst, 1784; *Elater aterrimus* Linnaeus, 1761 (as “aterrimus *Fab.*”); *Elater procerus* Illiger, 1807; *Elater longicollis* Olivier, 1790 (as “longicollis *Fab.*”); *Elater suturalis* Panzer, 1795; *Elater marginatus* Linnaeus sensu Paykull, 1800 (= *Elater longicollis* Olivier, 1790); *Elater haemorrhoidalis* Fabricius, 1801; *Elater ruficadis* Gyllenhal, 1808 (as “*ruficaudis Gyll.*”); *Elater inunctus* Panzer, 1795; *Elater vittatus* Fabricius, 1792; *Elater subfuscus* Gyllenhal, 1808.

Type species: *Elater vittatus* Fabricius, 1792 by subsequent designation (Ruiz 1996: 75).

Current status: junior objective synonym of *Athous* Eschscholtz, 1829 [Elateridae: Dendrometrinae: Dendrometrini: Dendrometrina] (*fide* Cate 2007: 156).

***Notopeda* Gistel, 1834b: 11**

Originally included available species: *Elater denticollis* Fabricius, 1794; *Elater linearis* Linnaeus, 1758 (as “linearis *id.* [Fab.]”; *Elater mesomelus* Linnaeus, 1758 (as “mesomelas *id.* [Fab.]”).

Type species: *Elater linearis* Linnaeus, 1758 by subsequent designation (Ruiz 1996: 110).

Current status: junior subjective synonym of *Denticollis* Piller & Mitterpacher, 1783 [Elateridae: Dendrometrinae: Dendrometrini: Denticollina] (*fide* Cate 2007: 167).

***Melanotus* Gistel, 1834b: 11**

Originally included available species: *Elater thoracicus* Fabricius, 1775; *Elater ruficollis* Linnaeus, 1758 (as “ruficollis *id.* [Fab.]”); *Elater discicollis* Herbst, 1806; *Elater biguttatus* Olivier, 1790 (as “biguttatus *Fab.*”); *Elater testaceus* Fabricius, 1792; *Elater equiseti* Herbst, 1784; *Elater lateralis* Fabricius, 1794; *Elater advena* Fabricius, 1787; *Elater rufipes* Fabricius, 1792.

Type species: *Elater thoracicus* Fabricius, 1775 (= *Elater gramineus* Scopoli, 1763) by subsequent designation (Ruiz 1996: 53).

Current status: junior homonym of *Melanotus* Erichson, 1829 (Coleoptera: Elateridae) and *Melanotus* Dejean, 1831 (Coleoptera: Carabidae); junior subjective synonym of *Cardiophorus* Eschscholtz, 1829 [Elateridae: Cardiophorinae] (*fide* Cate 2007: 194).

***Acanthus* Gistel, 1834b: 12**

Originally included available species: none. Therefore this name is a *nomen nudum*.

***Amphius* Gistel, 1834b: 12**

Originally included available species: *Elater riparius* Fabricius, 1792; *Elater rivularis* Gyllenhal, 1808; *Elater pulchellus* Linnaeus, 1761 (as “pulchellus *Fab.*”); *Elater quadripustulatus* Fabricius, 1792.

Type species: *Elater riparius* Fabricius, 1792 by subsequent designation (Ruiz 1996: 166).

Current status: junior objective synonym of *Hypnoidus* Dillwyn, 1829 [Elateridae: Dendrometrinae: Hypnoidini] (*fide* Cate 2007: 154).

***Trypheus* Gistel, 1834b: 12**

Originally included available species: *Elater elegans* Fabricius, 1792.

Type species: *Elater elegans* Fabricius, 1792 by monotypy.

Current status: junior subjective synonym of *Aeolus* Eschscholtz, 1829 [Elateridae: Agrypninae: ] (**new synonymy**).

***Epistrophus* Gistel, 1834b: 12**

Originally included available species: *Elater bimaculatus* Rossi, 1790 (as “bimaculatus *Fab.*”).

Type species: *Elater bimaculatus* Rossi, 1790 by monotypy.

Current status: junior objective synonym of *Drasterius* Eschscholtz, 1829 [Elateridae: Conoderinae: Conoderini] (*fide* Cate 2007: 105).

***Morous* Gistel, 1834b: 12**

Originally included available species: *Elater ferrugineus* Linnaeus, 1758 (as “ferrugineus *Fab.*”).

Type species: *Elater ferrugineus* Linnaeus, 1758 by monotypy.

Current status: junior objective synonym of *Elater* Linnaeus, 1758 [Elateridae: Elaterinae: Elaterini] (*fide* Cate 2007: 131).

***Antiphus* Gistel, 1834b: 12**

Originally included available species: *Elater plagiatus* Germar, 1823.

Type species: *Elater plagiatus* Germar, 1823 by monotypy.

Current status: junior subjective synonym of *Cardiorhinus* Eschscholtz, 1829 [Elateridae: Elaterinae: Agriotini: ] (**new synonym**).

***Anchialus* Gistel, 1834b: 12**

Originally included available species: none. Therefore this name is a *nomen nudum*.

***Lepidotus* Gistel, 1834b: 12**

Originally included available species: *Elater pilosus* Fabricius, 1792; *Elater vilis* Herbst, 1806 (as “vilis *Ill.*”); *Elater gilvellus* Dufour, 1824 (as “gilvellus *Zieg.*”); *Elater segetis* Bjerkander, 1779 (as “Segetis *Gyll.*”); *Elater variabilis* Fabricius, 1792; *Elater sputator* Linnaeus, 1758 (as “sputator *Fab.*”).

Type species: *Elater sputator* Linnaeus, 1758 by subsequent designation (Ruiz 1996: 129).

Current status: junior homonym of *Lepidotus* Asso, 1801 (Pisces) and *Lepidotus* Stephens, 1830 (Coleoptera: Elateridae); junior objective synonym of *Agriotes* Eschscholtz, 1829 [Elateridae: Elaterinae: Agriotini: Agriotina] (*fide* Cate 2007: 114).

***Amphilabris* Gistel, 1834b: 12**

Originally included available species: *Elater brunneus* Linnaeus, 1758 (as “brunneus *Fab.*”); *Elater fugax* Fabricius, 1801.

Type species: *Elater brunneus* Linnaeus, 1758 by subsequent designation (Ruiz 1996: 155).

Current status: junior objective synonym of *Sericus* Eschscholtz, 1829 [Elateridae: Elaterinae: Elaterini] (*fide* Cate 2007: 134).

***Eumenus* Gistel, 1834b: 12**

Originally included available species: *Elater theseus* Germar, 1817; *Elater aterrimus* Linnaeus, 1761; *Elater atratus* Illiger, 1805.

Type species: *Elater aterrimus* Linnaeus, 1761 by subsequent designation (Ruiz 1996: 140).

Current status: junior objective synonym of *Ectinus* Eschscholtz, 1829 [Elateridae: Elaterinae: Agriotini] (*fide* Cate 2007: 119).

***Phyletus* Gistel, 1834b: 12**

Originally included available species: *Cantharis sanguineus* Linnaeus, 1758 (as “sanguineus *Fab.*”).

Type species: *Cantharis sanguineus* Linnaeus, 1758 by monotypy.

Current status: junior objective synonym of *Lygistopterus* Dejean, 1833 [Lycidae: Lycinae: Calochromini] (**new synonym**).

***Homalysus* Gistel, 1834b: 12 (as “Homalysus*Ill.* ”)**

Note: This name is an unjustified emendation for *Omalisus* Geoffroy, 1762 [Omalisidae] and therefore a junior objective synonym of *Omalisus* Geoffroy, 1762.

***Phyllogaster* Gistel, 1834b: 13**

Originally included available species: *Lampyris laticornis* Fabricius, 1792.

Type species: *Lampyris laticornis* Fabricius, 1792 (= *Lampyris atra* Olivier, 1790) by monotypy.

Current status: junior subjective synonym of *Lucidota* Laporte, 1833 [Lampyridae: Lampyrinae: Lucidotini: Lucidotina] (**new synonym**).

***Pyrrhigius* Gistel, 1834b: 13**

Originally included available species: *Lampyris hemipterus* Geoffroy, 1762 (as “hemipterus *Fab.*”).

Type species: *Lampyris hemipterus* Geoffroy, 1762 by monotypy.

Current status: junior objective synonym of *Phosphaenus* Laporte, 1833 [Lampyridae: Lampyrinae: Lucidotini: ] (**new synonym**).

***Erota* Gistel, 1834b: 13**

Originally included available species: *Lampyris italica* Linnaeus, 1767 (as “italica *Fab.*”).

Type species: *Lampyris italica* Linnaeus, 1767 by monotypy.

Current status: junior objective synonym of *Luciola* Laporte, 1833 [Lampyridae: Luciolinae: Luciolini] (**new synonym**).

***Oxypterus* Gistel, 1834b: 13**

Originally included available species: *Lampyris maculata* DeGeer, 1774 (as “maculatus *Fab.*”).

Type species: *Lampyris maculata* DeGeer, 1774 by monotypy.

Current status: junior homonym of *Oxypterus* Rafinesque, 1814 (Mammalia) and *Oxypterus* Fleming, 1822 (Aves); junior subjective synonym of *Aspisoma* Laporte, 1833 [Lampyridae: Lampyrinae: Cratomorphini] (**new synonym**).

Note: Gistel (1834b: 13) also included “hesperae? *Fab.*” (= *Lampyris hespera* Linnaeus, 1767) and “4-punctulatus *Pty.*” The first species is conditionally included in the genus and therefore is not an originally included species (ICZN 1999: Article 67.2.5) and the second one is a *nomen nudum*.

***Phyllophagus* Gistel, 1834b: 13**

Originally included available species: *Telephorus xanthomelas* Perty, 1830 (as “xanthomelas. *Gistl. Pty.*”).

Type species: *Telephorus xanthomelas* Perty, 1830 by monotypy.

Current status: junior subjective synonym of *Chauliognathus* Hentz, 1830 [Cantharidae: Chauliognathinae: ] (**new synonym**).

Note: Gistel (1831: 307) described a new species under the name *Cantharis xanthomelas* which, based on Gistel’s (1834b: 13) entry, is a junior synonym of *Telephorus xanthomelas* Perty, 1830. The second species mentioned by Gistel (1834b: 13) under *Phyllophagus* (“dichrous *Pty.*”) is a *nomen nudum*.

***Epaphius* Gistel, 1834b: 13**

Originally included available species: *Dasytes variegatus* Germar, 1823; *Anobium lineatum* Fabricius, 1775; *Dasytes antis* Perty, 1830 (as “antis *id.* [Fab.]”).

Type species: *Dasytes variegatus* Germar, 1823 by **present designation**.

Current status: junior homonym of *Epaphius* Samouelle, 1819 (Coleoptera: Carabidae); senior subjective synonym of *Astylus* Laporte, 1836 [Melyridae: Melyrinae: ] (**new synonym**).

***Odontomerus* Gistel, 1834b: 13**

Originally included available species: none. Therefore this name is a *nomen nudum*.

***Isomerus* Gistel, 1834b: 14**

Originally included available species: *Choleva oblonga* Latreille, 1806.

Type species: *Choleva oblonga* Latreille, 1806 by monotypy.

Current status: junior subjective synonym of *Choleva* Latreille, 1797 [Leiodidae: Cholevinae: Cholevini: ] (**new synonym**).

***Berecyntha* Gistel, 1834b: 15**

Originally included available species: *Erotylus quadripustulatus* Fabricius, 1801.

Type species: *Erotylus quadripustulatus* Fabricius, 1801 by monotypy.

Current status: senior objective synonym of *Aulacochilus* Chevrolat, 1836 [Erotylidae: Erotylinae: Encaustini] (**new synonym**).

Note: Gistel’s name *Berecyntha* has not been used as a valid name after 1899 and *Aulacochilus* has been used for a particular taxon in at least 25 works, published by at least 10 authors in the immediately preceding 50 years and encompassing a span of not less than 10 years (see Appendix 1). Accordingly, following Article 23.9 (ICZN 1999), *Berecyntha* Gistel, 1834 is treated as a *nomen oblitum* and *Aulacochilus* Chevrolat, 1836 as a *nomen protectum*.

***Atomaria* Gistel, 1834b: 15**

Originally included available species: none. Therefore this name is a *nomen nudum*.

***Geophilus* Gistel, 1834b: 18**

Originally included available species: *Scarabaeus sabuleti* Panzer, 1797 (as “sabuleti *Fab.*”); *Scarabeus asper* Fabricius, 1775; *Scarabaeus porcatus* Fabricius, 1775; *Scarabaeus caesus* Creutzer, 1796 (as caesus *id.* [Fab.]”).

Type species: *Scarabeus asper* Fabricius, 1775 by **present designation**.

Current status: junior homonym of *Geophilus* Leach, 1814 (Chilopoda); junior objective synonym of *Psammodius* Fallén, 1807 [Scarabaeidae: Aphodiinae: Psammodiini] (**new synonym**).

***Ceraunus* Gistel, 1834b: 18**

Originally included available species: *Scarabaeus aegeon* Drury, 1773.

Type species: *Scarabaeus aegeon* Drury, 1773 by monotypy.

Current status: senior subjective synonym of *Golofa* Hope, 1837 [Scarabaeidae: Dynastinae: Dynastini] (**new synonym**).

Note: Gistel’s name *Ceraunus* has not been used as a valid name after 1899 and *Golofa* has been used for a particular taxon in at least 25 works, published by at least 10 authors in the immediately preceding 50 years and encompassing a span of not less than 10 years (see Appendix 2). Accordingly, following Article 23.9 (ICZN 1999), *Ceraunus* Gistel, 1834 is treated as a *nomen oblitum* and *Golofa* Hope, 1837 as a *nomen protectum*.

***Atrimedeus* Gistel, 1834b: 18**

Originally included available species: *Scarabaeus monodon* Fabricius, 1792; *Scarabaeus punctatus* Villers, 1789 (as “punctatus *id.* [Fab.]”).

Type species: *Scarabaeus punctatus* Villers, 1789 by **present designation.**

Current status: senior objective synonym of *Pentodon* Hope, 1837 [Scarabaeidae: Dynastinae: Pentodontini] (**new synonym**).

Note: Gistel’s name *Atrimedeus* has not been used as a valid name after 1899 and *Pentodon* has been used for a particular taxon in at least 25 works, published by at least 10 authors in the immediately preceding 50 years and encompassing a span of not less than 10 years (see Appendix 3). Accordingly, following Article 23.9 (ICZN 1999), *Atrimedeus* Gistel, 1834 is treated as a *nomen oblitum* and *Pentodon* Hope, 1837 as a *nomen protectum*.

***Eupalus* Gistel, 1834b: 18**

Originally included available species: *Scarabaeus retusus* Fabricius, 1781.

Type species: *Scarabaeus retusus* Fabricius, 1781 by monotypy.

Current status: junior objective synonym of *Coptorhinus* Dejean, 1833 but Dejean’s name was suppressed in Opinion 1838 (ICZN 1996); senior objective synonym of *Temnorhynchus* Hope, 1837 [Scarabaeidae: Dynastinae: Pentodontini] (**new synonym**).

Note: Gistel’s name *Eupalus* has not been used as a valid name after 1899 and *Temnorhynchus* has been used for a particular taxon in at least 25 works, published by at least 10 authors in the immediately preceding 50 years and encompassing a span of not less than 10 years (see Appendix 4). Accordingly, following Article 23.9 (ICZN 1999), *Eupalus* Gistel, 1834 is treated as a *nomen oblitum* and *Temnorhynchus* Hope, 1837 as a *nomen protectum*.

***Achates* Gistel, 1834b: 18**

Originally included available species: none. Therefore this name is a *nomen nudum*.

***Polycarmes* Gistel, 1834b: 18**

Originally included available species: *Melolontha orientalis* Krynicki, 1832 (as “orientalis *Zieg.*); *Melolontha australis* Gyllenhal, 1817 (as “australis *Schö.*”); *Melolontha pilosa* Fabricius, 1792; *Melolontha villosa* Fabricius, 1781.

Type species: *Melolontha villosa* Fabricius, 1781 by **present designation**.

Current status: junior objective synonym of *Anoxia* Laporte, 1832 [Scarabaeidae: Melolonthinae: Melolonthini] (**new synonym**).

Note: *Polycarmes* Gistel, 1834 is a senior homonym of *Polycarmes* Stål, 1867, currently a valid genus in Hemiptera (Pentatomidae). A new name is needed for the hemipteran generic name.

***Eurytes* Gistel, 1834b: 19**

Note: This name was proposed as a junior synonym of *Schizonycha* Dejean, 1833 [Scarabaeidae: Melolonthinae: Melolonthini] and not treated as valid subsequently. Therefore *Eurytes* Gistel, 1834 is a *nomen nudum*.

***Hylecharis* Gistel, 1834b: 19**

Originally included available species: none. Therefore this name is a *nomen nudum*.

***Phaedinus* Gistel, 1834b: 19**

Originally included available species: none. Therefore this name is a *nomen nudum*.

***Sogines* Gistel, 1834b: 19**

Originally included available species: none. Therefore this name is a *nomen nudum*.

***Demophila* Gistel, 1834b: 19**

Note: Gistel proposed this name for *Melolontha strigosa* Illiger, 1803 but in the errata (p. [36]) mentioned to replace *Demophila* with “Hymenoptia *Esch.*” (= *Hymenoplia* Eschscholtz, 1830) [Scarabaeidae: Melolonthinae: Sericini] and to add “H. bifrons *Esch.* Californ.” to this genus. Therefore Gistel’s *Demophila* is a *nomen nudum*.

***Nefaria* Gistel, 1834b: 19**

Originally included available species: none. Therefore this name is a *nomen nudum*.

***Acidota* Gistel, 1834b: 19**

Originally included available species: *Melolontha abdominalis* Fabricius, 1792.

Type species: *Melolontha abdominalis* Fabricius, 1792 by monotypy.

Current status: junior homonym of *Acidota* Stephens, 1829 (Coleoptera: Staphylinidae); junior objective synonym of *Amphicoma* Latreille, 1807 [Glaphyridae: ] (**new synonym**).

***Erymantis* Gistel, 1834b: 19**

Originally included available species: none. Therefore this name is a *nomen nudum*.

***Ostracodermus* Gistel, 1834b: 20**

Originally included available species: none. Therefore this name is a *nomen nudum*.

***Cecrops* Gistel, 1834b: 21**

Originally included available species: *Tenebrio gigas* Linnaeus, 1763; *Upis maxima* Germar, 1823.

Type species: *Tenebrio gigas* Linnaeus, 1763 by **present designation**.

Current status: junior objective synonym of *Mylaris* Pallas, 1781 [Tenebrionidae: Stenochiinae: Cnodalonini] (**new synonym**).

***Pythonissus* Gistel, 1834b: 21**

Originally included available species: *Helops morio* Fabricius, 1777; *Tenebrio elongatus* Palisot de Beauvois, 1817.

Type species: *Helops morio* Fabricius, 1777 (= *Tenebrio atratus* Fabricius, 1775) by **present designation**.

Current status: junior objective synonym of *Zophobas* Dejean, 1834 [Tenebrionidae: Tenebrioninae: Tenebrionini] (**new synonym**).

***Physignathus* Gistel, 1834b: 22**

Originally included available species: *Helops undatus* Fabricius, 1792.

Type species: *Helops undatus* Fabricius, 1792 (= *Erotylus nebulosus* Fabricius, 1781) by monotypy.

Current status: junior homonym of *Physignathus* Cuvier, 1829 (Reptilia); junior objective synonym of *Cymatothes* Dejean, 1834 [Tenebrionidae: Tenebrioninae: Amarygmini] (**new synonym**).

***Pelops* Gistel, 1834b: 22**

Originally included available species: *Helops ater* Fabricius, 1775.

Type species: *Helops ater* Fabricius, 1775 by monotypy.

Current status: senior objective synonym of *Prionychus* Solier, 1835 [Tenebrionidae: Alleculinae: Alleculini: Alleculina] (**new synonym**).

Note: Gistel’s name *Pelops* has not been used as a valid name after 1899 and *Prionychus* has been used for a particular taxon in at least 25 works, published by at least 10 authors in the immediately preceding 50 years and encompassing a span of not less than 10 years (see Appendix 5). Accordingly, following Article 23.9 (ICZN 1999), *Pelops* Gistel, 1834 is treated as a *nomen oblitum* and *Prionychus* Solier, 1835 as a *nomen protectum*.

***Gerontes* Gistel, 1834b: 27**

Note: This name was proposed as a junior synonym of *Mecocorhynus* Schönherr, 1825 [Curculionidae: Molytinae: Ithyporini: ] and not treated as valid subsequently. Therefore *Gerontes* Gistel, 1834 is a *nomen nudum* as mentioned by Alonso-Zarazaga and Lyall (1999: 203).

***Gymnotrion* Gistel, 1834b: 27**

Originally included available species: none. Therefore this name is a *nomen nudum*.

***Ceratias* Gistel, 1834b: 28**

Originally included available species: none. Therefore this name is a *nomen nudum*.

***Ceratades* Gistel, 1834b: 29**

Originally included available species: *Cerambyx sutor* Linnaeus, 1758; *Lamia sartor* Fabricius, 1787.

Type species: *Cerambyx sutor* Linnaeus, 1758 by **present designation**.

Current status: junior objective synonym of *Monochamus* Dejean, 1821 [Cerambycidae: Lamiinae: Monochamini] (*fide* Adlbauer et al. 2010: 281).

***Dendrobius* Gistel, 1834b: 30**

Originally included available species: *Cerambyx curculionoides* Linnaeus, 1760.

Type species: *Cerambyx curculionoides* Linnaeus, 1760 by monotypy.

Current status: junior homonym of *Dendrobius* Meyen, 1833 (Mammalia); junior objective synonym of *Mesosa* Latreille, 1829 [Cerambycidae: Lamiinae: Mesosini] (*fide* Adlbauer et al. 2010: 272).

***Eurypedus* Gistel, 1834b: 31**

Originally included available species: *Cassida oblonga* Thon, 1826.

Type species: *Cassida oblonga* Thon, 1826 by monotypy.

Current status: valid genus [Chrysomelidae: Cassidinae: Ischyrosonychini] (*fide* Shin 2016: 329).

***Accantosomus* Gistel, 1834b: [36**]

Originally included available species: *Elater ligneus* Linnaeus, 1763 (as “ligneus *Fab.*”).

Type species: *Elater ligneus* Linnaeus, 1763 by monotypy.

Current status: junior subjective synonym of *Semiotus* Eschscholtz, 1829 [Elateridae: Semiotinae] (**new synonym**).

## Appendix 1

References supporting the conservation of *Aulacochilus* Chevrolat, 1836 [Erotylidae] over *Berecyntha* Gistel, 1834 through the reversal of precedence.

Chûjô M (1968) Erotylid beetles from south-China, Hainan, Taiwan and the Ryukyus. Studies on the erotylid beetles (20). Pacific Insects 10: 539–550.

Chûjô M (1968) Erotylid beetles from Thailand, Laos and Viet-Nam. Pacific Insects 10: 551–573.

Chûjô M (1969) Erotylidae (Insecta: Coleoptera). Fauna Japonica. Academic Press of Japan, Tokyo. xii + 316 pp.

Chûjô M (1974) Erotylid-beetles from Thailand, Malaysia, Sarawak and Philippines. Studies on the erotylid-beetles (22). Bulletin of the Japan Entomological Academy 9: 1–19.

Chûjô M (1975) Coleoptera: Erotylidae and Languriidae from Ceylon. Entomologica scand (Suppl. ) 4: 195–198.

Chûjô M (1987) Erotylidae from New Guinea and her adjacent islands I (Coleoptera). Esakia 25: 5–36.

Chûjô M, Chûjô M (1988) A catalog of the Erotylidae (Insecta, Coleoptera) from the Old World (excl. the Ethiopian Region). Esakia 26: 139–185.

Chûjô M, Chûjô M, Lee CE (1993) Erotylidae from Korea (Insecta, Coleoptera). Esakia 33: 99–108.

Drilling K, Dettner K, Klass K-D (2013) The distribution and evolution of exocrine compound glands in Erotylinae (Insecta: Coleoptera: Erotylidae). Annales de la Société Entomologique de France (Nouvelle Série) 49: 36–52. https://doi.org/10.1080/00379271.2013.763458

Iablokoff-Khnzorian SM (1975) Etude sur les Erotylidae (Coleoptera) palearctiques. Acta Zoologica Cracoviensia 20: 201–249.

Jung SH, Oh SH (2012) Insect Fauna of Yeongsil in Mt. Hallasan National Park (excluding Lepidoptera). Journal of Korean Nature 5: 27–36. https://doi.org/10.7229/jkn.2012.5.1.027

Klimov PB (2000) A description of a new genus, *Umakefeq* gen. n., including three new species of mycetophagous acarid mites (Acariformes, Acaridae) from eastern Palaearctic. Acarina 7: 93–106.

Kobayashi T, Sota T (2016) Distance decay of similarity in fungivorous insect communities: assessing dispersal limitation using genetic data. Ecosphere 7(6): 1–11. https://doi.org/10.1002/ecs2.1358

Lafer GS (2000) *Lioptera erotyloides* Bates, 1883 newly recorded species of the ground beetles (Coleoptera: Carabidae, Lebiinae) from South Korea. Far Eastern Entomologist 91: 11–12.

Lawrence JF (1988) Notes on the classification of some Australian Cucujoidea (Coleoptera). Journal of Australian Entomological Society 27: 53–54. https://doi.org/10.1111/j.1440-6055.1988.tb01144.x

Lawrence JF, Ślipiński A (2013) Australian beetles: morphology, classification and keys. Volume 1. CSIRO Publishing, Collingwood.

Li J, Ren GD, Zhao X (2013) Two new record species of the genus *Aulacochilus* (Coleoptera: Erotylidae) from China. Journal of Hebei University Natural Science Edition 33: 520–523.

Lim JS, Lee BW, Park SY, Jo DG (2011) Insect fauna of Maebongsan Mountain, Hongcheon-gun, Gangwon-do. Journal of Korean Nature 4: 293–307. https://doi.org/10.7229/jkn.2011.4.4.293

Pal TK (1993) On a collection of clavicorn beetles (families Erotylidae, Endomychidae and Languriidae) from Arunachal Pradesh, India. Records of the Zoological Survey of India 90 [1992]: 203–210.

Pal TK (2003) Insecta: Coleoptera: Erotylidae. Pp. 99–106 *in*: Alfred JRB (Ed) Fauna of Sikkim (part-3). State Fauna Series 9. Zoological Survey of India, Kolkata, 411 pp.

Robertson JA, McHugh JV, Whiting MF (2004) A molecular phylogenetic analysis of the pleasing fungus beetles (Coleoptera: Erotylidae): evolution of colour patterns, gregariousness and mycophagy. Systematic Entomology 29: 173–187. https://doi.org/10.1111/j.0307-6970.2004.00242.x

Sasakawa M, Negishi T (1973) Laboratory and field studies on behaviouristic response of the minute pine beetle, *Cryphalus fulvus* Niijima (Coleoptera: Scolytidae) to the female pheromone. Applied Entomology and Zoology 8: 143–156.

Setsuda K (1993) The component and structure of the beetle community inhabiting fruit bodies of wood-rotting fungi. Akitu (Suppl. No. 1): 1–21.

Skelley PE (1998) A catalogue of the Crotch collection of Erotylidae (Coleoptera). Annales Zoologici 48: 1–44.

Wêgrzynowicz P (2002) Morphology, phylogeny and classification of the family Erotylidae based on adult characters (Coleoptera: Cucujoidea). Genus 13: 435–504.

Wêgrzynowicz P (2007) Family Erotylidae Latreille, 1802. Pp. 531–546 *in*: Löbl I, Smetana A (Eds) Catalogue of Palaearctic Coleoptera. Volume 4. Elateroidea–Derodontoidea–Bostrichoidea–Lymexyloidea–Cleroidea–Cucujoidea. Apollo Books, Stenstrup, 935 pp.

Zaitsev AA, Kompantsev AV, Zaitsev AI (2016) A review of larval Encaustini (Coleoptera: Erotylidae) from Russia. Russian Entomological Journal 25: 367–386.

## Appendix 2

References supporting the conservation of *Golofa* Hope, 1837 [Scarabaeidae] over *Ceraunus* Gistel, 1834 through the reversal of precedence.

## Appendix 3

References supporting the conservation of *Pentodon* Hope, 1837 [Scarabaeidae] over *Atrimedeus* Gistel, 1834 through the reversal of precedence.

## Appendix 4

References supporting the conservation of *Temnorhynchus* Hope, 1837 [Scarabaeidae] over *Eupalus* Gistel, 1834 through the reversal of precedence.

## Appendix 5

References supporting the conservation of *Prionychus* Solier, 1835 [Tenebrionidae] over *Pelops* Gistel, 1834 through the reversal of precedence.
